# Influenza Vaccination in Adults in the United States with COPD before and after the COVID-19 Pandemic (2017–2022): A Multi-Year Cross-Sectional Study

**DOI:** 10.3390/vaccines12080931

**Published:** 2024-08-21

**Authors:** Marissa Wold, Sanda Cristina Oancea

**Affiliations:** Master of Public Health Program, Department of Population Health, School of Medicine and Health Sciences, University of North Dakota, Grand Forks, ND 58202, USA; woldx173@umn.edu

**Keywords:** COPD, chronic obstructive pulmonary disease, emphysema, chronic bronchitis, influenza vaccination, flu shot, flu vaccine, COVID-19, BRFSS

## Abstract

There is limited literature regarding seasonal influenza vaccination (SIV) among those with a history of chronic obstructive pulmonary disease (HCOPD) prior to the COVID-19 pandemic, and no information on the topic assessing the years following the pandemic. This cross-sectional study used the Behavioral Risk Factor Surveillance Survey (BRFSS) data from the years 2017 to 2022 (*n* = 822,783 adults ages 50–79 years; 50.64% males). The exposure was a HCOPD, and the outcome was SIV within the past year. Weighted and adjusted logistic regression models were conducted overall and by the significant effect modifiers: smoking status, sex, and year. Having an HCOPD significantly increases the weighted adjusted odds (WAO) of SIV when compared to not having an HCOPD overall and by smoking status, sex, and year. For 2017 through 2022, among all current, former, and never smokers with an HCOPD, the WAO of SIV were: 1.36 (1.28, 1.45), 1.35 (1.27, 1.43), and 1.18 (1.09, 1.27), respectively. Among males with an HCOPD who were current, former, and never smokers, the WAO of SIV were: 1.35 (1.23, 1.48), 1.45 (1.33, 1.58), and 1.23 (1.05, 1.44), respectively. Among females with an HCOPD who were current, former, and never smokers, the WAO of SIV were: 1.31 (1.20, 1.43), 1.24 (1.15, 1.35), and 1.13 (1.04, 1.23), respectively. Study findings suggest males had significantly greater WAO ratios of receiving SIV than females in 2020 and 2022, during and after the COVID-19 pandemic. More specifically, males with an HCOPD who were former smokers had significantly greater WAOR of receiving SIV than females in 2020 and 2022. Understanding the potential barriers to SIV receipt by smoking status and sex, especially during a pandemic, and especially for individuals impacted by an HCOPD, is essential for better health interventions in times of a national crisis such as a pandemic. Additionally, SIV receipt is low among those with an HCOPD, and efforts should be made to improve this.

## 1. Introduction

Chronic obstructive pulmonary disease (COPD), comprising chronic bronchitis and emphysema, affects nearly 16 million Americans and is the fourth-leading cause of age-adjusted death in the US [1]. The average COPD patient experiences between 1 and 4 acute exacerbations annually; as a result of these exacerbations, there are over 700,000 total COPD-related hospital stays in the US on average each year [2]. Increased frequency and severity of exacerbation events are associated with poorer COPD prognosis, including more rapid disease progression and greater risk of mortality [3,4]. The risk of nosocomial (hospital-acquired) infections, though these account for a comparatively low proportion of annual influenza cases, should also be considered when discussing COPD exacerbation and complications [5]. The decline in overall health and quality of life following a COPD exacerbation places an individual at increased risk of future exacerbation and readmission [6]. Exacerbation is the most common cause of hospitalization among COPD patients, and the etiology is most often infectious [4]. It is estimated around half of these infections in US adults with a history of COPD are due to viral causes, including influenza and COVID-19 [7,8]. The remaining half of infectious exacerbation originates from bacterial species, most commonly *M. catarrhalis* and *S. pneumoniae* [9]. There is also an established link between influenza A and B infections and a predisposition to secondary infection leading to bacterial pneumonia, making influenza vaccination a chief concern for sufferers of COPD [10,11].

The influenza vaccination has been demonstrated in multiple clinical and observational studies to exert a protective effect against contraction and subsequent death from the resulting exacerbation in individuals with a history of COPD [12,13,14]. Additionally, the risk–benefit ratio for influenza vaccination in this population is favorable [15]. Because of this, major medical organizations within the US, including the Centers for Disease Control (CDC) and the National Institutes of Health (NIH), endorse an annual flu vaccine for the COPD population.

The current literature has limited information regarding influenza vaccination among individuals with a history of COPD (HCOPD) prior to the COVID-19 pandemic, and no information on the topic assessing the years following the pandemic. The majority of the literature prior to the pandemic assesses the safety and efficacy of the influenza vaccination in COPD patients, rather than focusing on an examination of actual vaccination receipt. Little is known about influenza vaccination in persons with a history of COPD in the years following the 2020 pandemic. Americans have demonstrated deteriorating attitudes toward vaccination in general following lockdowns, mask mandates, and politicization of this public health issue, and as a result, the general population may be more likely to decline influenza vaccination [16,17]. However, it is unknown how the pandemic has impacted the influenza vaccination behavior of US adult individuals with an HCOPD.

The present study aims to examine the association between HCOPD and influenza vaccination among US adults and to answer the following research questions:

1. Are US adults with an HCOPD more likely to receive seasonal influenza vaccination than adults without an HCOPD?
2. Has this relationship changed over time, and was it impacted by the COVID-19 pandemic?
3. Are there any effect modifiers of this association?

We hypothesize that the odds of receiving a seasonal influenza vaccination will be higher among US adults with an HCOPD compared to adults without an HCOPD. We further hypothesize that this vaccine-seeking behavior changed among both populations with regard to the years prior to, during, and following the 2020 pandemic.

## 2. Methods

### 2.1. Dataset

The Behavioral Risk Factor Surveillance Survey (BRFSS) is an annual telephone survey conducted by the CDC in all 50 states, the District of Columbia, and 3 participating US territories [18]. It collects demographic and health data from over 400,000 US adults each year, including information on chronic illness, access to care, prevention, and health risk behaviors. The data used for this study is extracted from BRFSS 2017 through BRFSS 2022.

### 2.2. Exposure of Interest: History of COPD

The exposure variable is having a history of chronic obstructive pulmonary disease (HCOPD), comprising chronic bronchitis and emphysema. This is a dichotomous variable with possible answers of “Yes” or “No” to the following question: “Have you ever been told you have chronic obstructive pulmonary disease, COPD, emphysema, or chronic bronchitis?”. Individuals who answered with “Don’t know/not sure”, “Refused”, or those with missing values for this question were excluded from this study.

### 2.3. Outcome of Interest: Receipt of Annual Influenza Vaccination

The outcome variable is the receipt of seasonal influenza vaccine (SIV) within the 12 months prior to the survey as reported by the subject. This is also a dichotomous variable, with possible answers of “Yes” or “No” to the following question: “During the past 12 months, have you had either a flu shot or flu vaccine that was sprayed in your nose?”. Individuals who answered with “Don’t know/not sure”, “Refused”, or those with missing values for this question were excluded from this study.

### 2.4. Confounders of Interest

**Figure 1:**
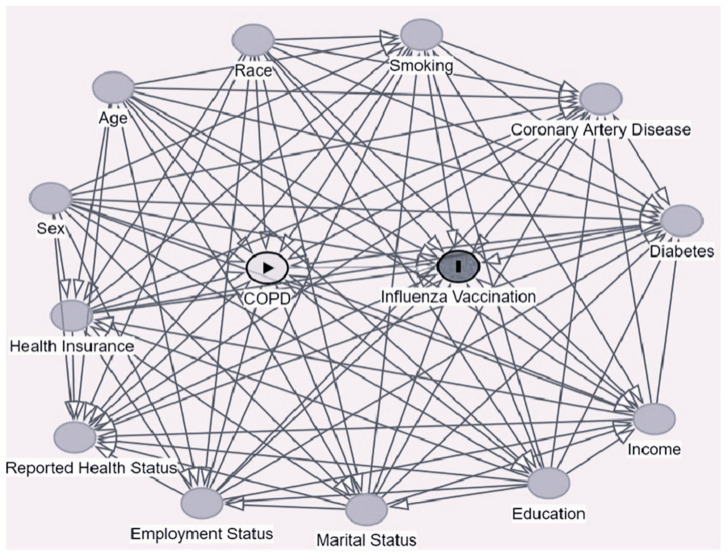
Directed acyclic graph (DAG).

The confounders of interest used in this study were selected based on a review of the previous literature; they were identified to be risk factors for the outcome and the exposure or were associated with (but not a result of) the exposure under investigation. Following the identification of the confounders of interest, they were included in a directed acyclic graph (DAG), and the minimum sufficient set of confounders was determined thereof (Figure 1). The final set of confounders included in this study included age [19,20,21,22,23], race [21,24], sex [21,22,23], education level [21,25,26], employment status [22,24], income level [21,22,23,27], health insurance [23,28], marital status [20,25,26], self-reported health status [29,30], coronary artery disease status [23,25,26,28], diabetes mellitus status [25,26,28], and smoking history [31,32].

### 2.5. Final Study Sample

**Figure 2:**
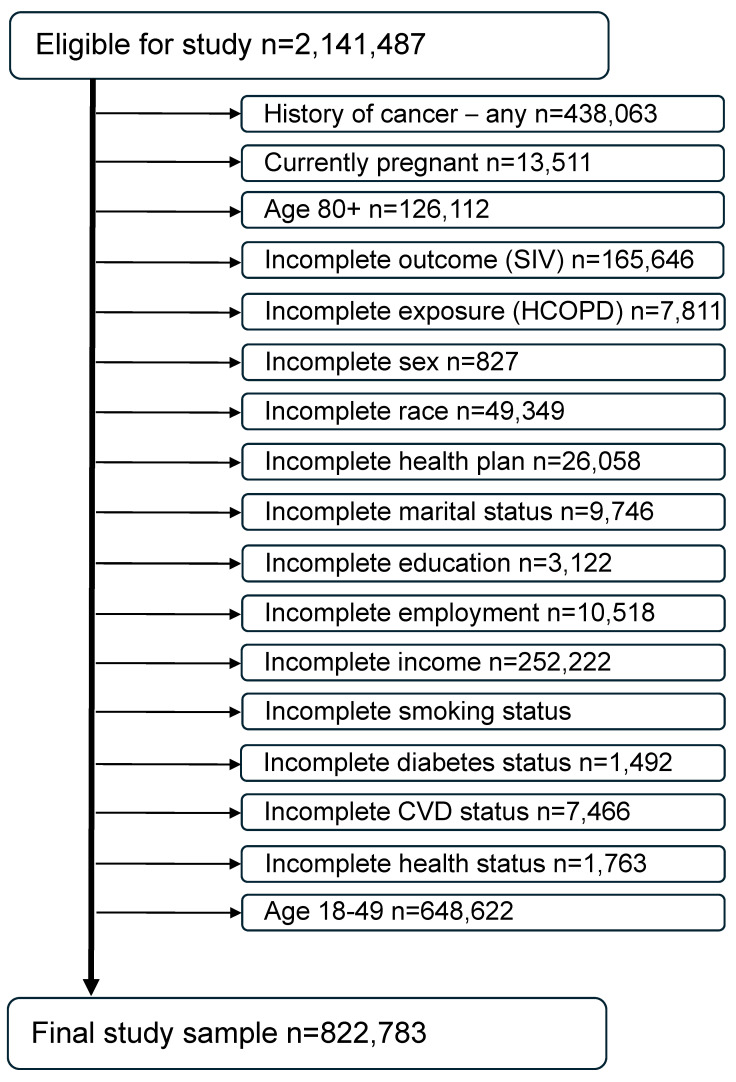
Consort diagram of the final study sample (combined BRFSS years 2017 through 2022).

For the 6 years under investigation (2017–2022), a total of 2,591,503 records were available. Individuals aged 80 and older were excluded because they cannot be assessed by years of age since the BRFSS imputes all of their ages to 80. Individuals under age 50 were excluded for being unlikely to have an HCOPD and therefore to minimize any age effect on results. Women who were pregnant at the time of the survey were also excluded because of their physiologic differences from the nonpregnant population of adults [33]. Individuals with a previous history of cancer were excluded from this study since they may be immunocompromised due to their cancer treatment or in a delicate state post-treatment and therefore more encouraged by their healthcare provider to receive the SIV [34,35]. In addition, individuals with missing data or answers of “Don’t know/not sure” on any of the exposure, outcome, or confounders of interest included in the models were also excluded from the analyses. The final study sample size is 822,783 (Figure 2).

### 2.6. Statistical Analyses

Based on the DAG, the confounders included in the analysis were age, race, education, employment, income, marital status, self-reported health status, health insurance, coronary artery disease, diabetes, and smoking status. Descriptive statistics based on these confounding variables of interest were computed. Multiple weighted unadjusted and weighted adjusted logistic regression models were conducted to assess the association between an HCOPD and receipt of SIV. The effect modifying the role of several variables of interest was investigated and, when found to be significant, subgroup analyses were performed. All statistical analyses were performed using SAS version 9.4 (SAS Institute, Cary, NC, USA) and SAS OnDemand for Academics (SAS Institute, Cary, NC, USA). SAS survey procedures (including SURVEYMEANS, SURVEYFREQ, and SURVEYLOGISTIC) were utilized to account for the complex sampling design of the BRFSS. Statistical significance for all analyses was set to *p* < 0.05.

## 3. Results

### 3.1. Characteristics of the Final Study Sample

Overall, 49.36% (95% confidence interval (CI): 49.11, 49.61) of US adults ages 50–79 years in the final study sample (n = 822,783) had the outcome under investigation (receipt of seasonal influenza vaccination (SIV) in the past 12 months). The prevalence of the outcome was significantly greater in individuals with an HCOPD compared to those without an HCOPD, 54.80% (95% CI: 54.04, 55.56) versus 48.79% (95% CI: 48.53, 49.05), respectively (Table 1). Within the final study sample, 9.44% (95% CI: 9.30, 9.58) of US adults had the exposure under investigation (HCOPD). People who received SIV were older than those who did not (median age 62.16 versus 58.62, respectively). However, among people with healthcare coverage, 48.76% (95% CI: 48.50, 49.01) did not receive SIV. Overall, men were significantly less likely (46.70%; 95% CI: 46.35, 47.05) to be vaccinated against influenza than women (51.95%; 95% CI: 51.60, 52.29). Study participants identifying as white were significantly more likely to have received SIV than all other races except Asian (50.52%; 95% CI: 50.27, 50.79 versus 55.38%; 95% CI: 53.31, 57.46, respectively). People with an income of $50,000 or more were significantly more likely to have received SIV (52.44%; 95% CI: 52.11, 52.78) than all other income groups. Never smokers were significantly more likely to have received SIV (50.43%; 95% CI: 50.09, 50.78) compared to current smokers (37.42%; 95% CI: 36.84, 38.01), but less likely than former smokers (53.28%; 95% CI: 52.85, 53.71). People with other chronic conditions like coronary heart disease (58.56%; 95% CI: 57.67, 59.45 versus 48.72%; 95% CI: 48.46, 48.98) and diabetes (57.70%; 95% CI: 57.12, 58.29 versus 47.44%; 95% CI: 47.17, 47.72) were significantly more likely to have received SIV than those who did not have either condition.

### 3.2. Weighted and Adjusted Logistic Regression Results

The overall weighted and adjusted odds (WAO) of receiving SIV in the past 12 months among US adults with a history of COPD (HCOPD) were 23% greater (95% CI: 1.19, 1.28) than the WAO of SIV among US adults without an HCOPD (Table 2).

For the years 2017–2022, among men with an HCOPD (8.51%), and among women with an HCOPD (10.34%), the WAO (95% CI) of SIV were: 1.30 (1.13, 1.49); 1.28 (1.13, 1.45), 1.59 (1.40, 1.80); 1.62 (1.34, 1.96); 1.16 (1.01, 1.34); and 1.39 (1.22, 1.58); 1.35 (1.21, 1.51); 1.28 (1.14, 1.43); 1.24 (1.11, 1.38); 1.16 (1.01, 1.33); 1.16 (1.02, 1.31); and 1.26 (1.12, 1.42) when compared to men and women without an HCOPD, respectively.

**Figure 3:**
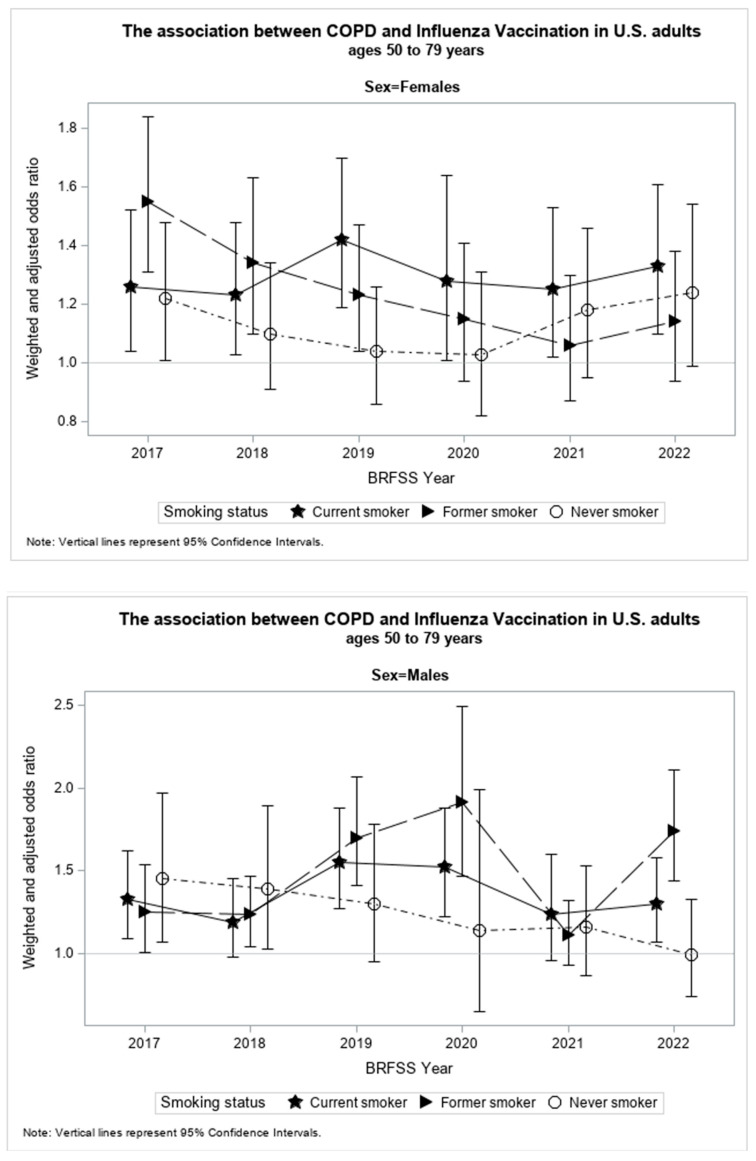
WAO with 95% confidence intervals by year of investigation for females and males of each smoking status.

The present study found smoking status, sex, and the study year of investigation to be significant effect modifiers (*p* < 0.05) of the association between an HCOPD and SIV. Table 2 shows results from the stratified weighted and adjusted logistic regression models by smoking status, sex, and study year. Figure 3 illustrates the relationship between WAORs and their corresponding 95% confidence intervals (CI) for each study subgroup in graphical form. The increase or decrease in WAOR and 95% CI by study year of investigation for each smoking status is displayed for females and males.

Among current smokers, males with an HCOPD had 35% significantly greater WAO (95% CI: 1.23,1.48) to receive SIV than males without an HCOPD. The WAO of receiving SIV among currently smoking men was significantly, or marginally significantly, greater in those who had an HCOPD compared to their counterparts without an HCOPD for all the years under investigation (Table 2).

Among currently smoking females with an HCOPD, the WAO of receiving SIV was 31% (95% CI: 1.20, 1.43) significantly greater than among their counterparts without an HCOPD for all the years under investigation.

Overall, although male current smokers had a greater WAOR of receiving SIV than female current smokers, this difference was not statistically significant.

Among former smokers, males with an HCOPD had 45% significantly greater WAO (95% CI: 1.33, 1.58) to receive SIV than males without an HCOPD. This association was significant for all the years under investigation, except for 2021, the second year of the COVID-19 pandemic.

Among former smokers, females with an HCOPD had 24% significantly greater WAO (95% CI: 1.15, 1.35) to receive SIV than females without an HCOPD. This association was significant for all the years under investigation, with the exception of 2020, 2021, and 2022, the years of the COVID-19 pandemic.

Overall, although male former smokers had a greater WAOR of receiving SIV than female current smokers, this difference was not statistically significant.

Among never smokers, males with an HCOPD had 23% significantly greater WAO (95% CI: 1.05, 1.44) of receiving SIV than males without an HCOPD. This association was only significant in 2017 and 2018 among all years under investigation.

Among never smokers, females with an HCOPD had 13% times significantly greater WAO (95% CI: 1.04, 1.23) to receive SIV than females without an HCOPD. This association was only marginally significant, or significant, in 2017 and 2022 among all years under investigation.

Overall, although male never smokers had greater WAOR of receiving SIV than female never smokers, this difference was not statistically significant.

## 4. Discussion

A significant positive association was found between having an HCOPD and receiving SIV among US adults from 2017 to 2022. This association was greater among current and former smokers who were males when compared to their female counterparts, but not significantly different among never smoker males and females.

The overall observed positive association between having an HCOPD and SIV receipt is unsurprising given that infection with influenza has long been a concern for populations highly susceptible to respiratory infection such as persons with an HCOPD [36,37]. As one of the most common viral causes of COPD exacerbation, influenza is an important topic of consideration in the management of those with an HCOPD. Despite the positive association between an HCOPD and receipt of SIV, nearly half of the individuals with an HCOPD in this study reported not having had SIV within the past 12 months. Though in many of the examined subgroups, a person with an HCOPD was significantly more likely than a counterpart without an HCOPD to have received SIV, there is insufficient coverage for adequate prevention among this population. A multitude of factors may be involved in producing this observation given the complex nature of vaccine acceptance and general attitudes toward vaccination among US adults in recent years.

The recent COVID-19 pandemic exerted many far-reaching effects on the US population, including a decline in attitude toward vaccinations, including SIV [16,17]. One longitudinal study which began in the early days of COVID-19’s spread to the United States found that, by August 2020, there was a significant decline in general attitude toward vaccination as well as a decrease in SIV intention for the coming season [16]. Age, socioeconomic status, and political affiliation were determined to play a role in this change [16]. A different longitudinal study beginning early in the spread of COVID-19 in the US demonstrated that US women reported higher levels of fear and stress associated with the pandemic than men and generally had a higher perception of the severity of the situation and, as a result, exhibited greater adherence to preventive measures [38]. This could be one explanation for the lower odds of SIV in those with an HCOPD that were observed in female former smokers in 2020, 2021, and 2022. It is possible that in this population, concern for COVID-19 risk outweighed the desire for influenza prevention.

A study examining BRFSS data from 2008 found that current smokers were overall less likely to receive SIV than former or never smokers; of all three groups, former smokers were most likely to receive SIV [39]. This is consistent with our overall results, which demonstrate that, irrespective of an HCOPD, former smokers are most likely to have received SIV, followed by never smokers, then current smokers. When an HCOPD is considered, there is a significant difference between former smokers and never smokers of both sexes overall, though this relationship does not persist by individual year. This finding may be due to awareness of increased risk and resultant increased interest in preventive behaviors among some people with an HCOPD. Previous research conducted internationally has found that the existence of chronic health conditions may increase an individual’s likelihood of influenza vaccination [15,29].

SIV behaviors of US adults with an HCOPD prior to the pandemic have previously been poorly described, with the majority of the literature on the topic originating outside the United States. These international studies identified several barriers to SIV among adults with an HCOPD, including individual concerns regarding SIV safety in the HCOPD population, lack of education regarding the necessity and efficacy of SIV in persons with an HCOPD, and lack of healthcare provider recommendation for SIV [40,41,42,43]. US-based studies involving SIV in those with an HCOPD are limited. Therefore, the current study may provide some insight to inform efforts to improve SIV rates among US adults with HCOPD in the future, but longitudinal research is needed. This suggestion is predicated on the diagnosis of COPD having been previously made in an individual. There is concern for underdiagnosis of COPD, which, of course, has the potential to further decrease the rate of SIV receipt in those most affected [44].

The main limitation of this study is that one cannot assess causality given the cross-sectional nature of the design, which does not allow for any temporal consideration like risk. There are some other limitations inherent in the BRFSS data, such as the self-reported nature of the data, the lack of questions regarding beliefs and attitudes, and the inability to generalize beyond the United States. Another limitation is that there are an unknown number of people living with COPD in the US who have never been diagnosed. One estimate based on 2007–2010 NHANES data suggested there may be as many as 28.9 million Americans living with some form of obstructive lung disease, which suggests that there is severe underdiagnosis of COPD [44]. However, the majority of that estimate (15.9 million) was suggested to have some form of mild obstructive disease, and that study was not limited to COPD, instead including any obstructive lung disease in a population aged 20–79 [44]. Finally, due to the confines of BRFSS contents, this analysis is unable to account for all possible comorbidities an individual may have.

The most obvious strengths of this study are the very large sample size and national representation of the data. With an initial pool of over 2 million respondents, this study was able to analyze the data from over 800,000 survey respondents across a span of 6 years. The years under investigation, representing three years prior to the COVID-19 pandemic and three years from the beginning of the pandemic, can provide insights into how behaviors might have changed during that trying time. Additionally, this study is the first of its kind, representing a large, multi-year, nationwide analysis examining SIV behaviors before, during, and following the COVID-19 pandemic. The analysis itself is also robust, being the best fit for the type of data and study design. Finally, the BRFSS itself exhibits strong validity and reliability as compared with other national surveys and is thus an excellent source of data [45].

## 5. Conclusions

Influenza prevention through improved influenza vaccination rates among individuals with an HCOPD will reduce the number of annual COPD exacerbations, thus improving the quality of life for these individuals and decreasing the overall economic burden of COPD-exacerbation-related hospitalization. Enhanced educational initiatives and promotional efforts by public health officials regarding the importance of influenza vaccination for high-risk groups, such as individuals with an HCOPD, could substantially improve health outcomes and quality of life for this population of nearly 16 million Americans, particularly during national crises like a pandemic. Special consideration should be given to current and former smokers, and the impact of sex differences on this association warrants further investigation through longitudinal study designs. Furthermore, enhanced efforts to identify and diagnose COPD are warranted.

## Figures and Tables

**Table 1:** Descriptive statistics for US adults aged 50–79, 2017 through 2022.

	Overall (N = 822,783)			Received SIV Past 12 Months				*p* Value
				Yes (N = 431,990)		No (N = 390,793)		
	Unweighted Counts	Weighted		Weighted		Weighted		
		Median	IQR	Median	IQR	Median	IQR	
Age at survey	822,783	60.25	54.36–66.89	62.16	55.86–68.84	58.62	53.40–64.55	<0.0001
	Overall (N = 822,783)			Received SIV past 12 months				*p* value
				Yes (N = 431,990)		No (N = 390,793)		
	Unweighted counts	Weighted		Weighted		Weighted		
		Percent	95% CI for percent	Percent	95% CI for percent	Percent	95% CI for percent	
Ever Diagnosed with COPD								
*Yes*	80,710	9.44	9.30–9.58	54.80	54.04–55.56	45.20	44.44–45.96	<0.0001
*No*	742,073	90.56	90.42–90.70	48.79	48.53–49.05	52.21	50.95–51.47	
Healthcare Coverage								
*Yes*	783,275	93.70	93.56–93.84	51.24	50.99–51.50	48.76	48.50–49.01	<0.0001
*No*	39,508	6.30	6.16–6.44	21.32	20.47–22.17	78.68	77.83–79.53	
Sex								
*Male*	379,462	50.64	50.39–50.89	46.70	46.35–47.05	53.30	52.95–53.65	<0.0001
*Female*	443,321	49.36	49.12–49.61	51.95	51.60–52.29	48.05	47.71–48.40	
Race								
*White, non-Hispanic*	682,856	76.56	76.31–76.80	50.52	50.27–50.79	49.47	49.21–49.73	<0.0001
*Black, non-Hispanic*	71,355	12.88	12.70–13.06	44.29	43.55–45.04	55.71	54.96–56.45	
*American Indian/Alaska Native, non-Hispanic*	16,629	1.58	1.52–1.64	42.61	40.84–44.38	57.39	55.62–59.16	
*Asian, non-Hispanic*	15,357	4.26	4.09–4.43	55.38	53.31–57.46	44.62	42.54–46.69	
*Other races*	34,607	4.73	4.61–4.85	41.16	39.85–42.46	58.84	57.54–60.15	
Income								
*<$15,000*	70,276	9.39	9.24–9.54	41.56	40.72–42.41	58.44	57.60–59.28	<0.0001
*$15,000 to <$25,000*	111,307	13.50	13.32–13.67	44.64	43.96–45.31	55.37	54.70–56.04	
*$25,000 to <$35,000*	86,396	10.06	9.91–10.21	46.85	46.07–47.63	53.15	52.37–53.93	
*$35,000 to <$50,000*	113,246	12.59	12.43–12.75	48.89	48.21–49.58	51.11	50.42–51.79	
*$50,000 or more*	441,567	54.46	54.21–54.71	52.44	52.11–52.78	47.56	47.22–47.89	
Employment Status								
*Employed for wages/self-employed*	389,833	50.71	50.46–50.96	41.43	40.50–42.35	58.56	57.65–59.50	<0.0001
*Homemaker/student/retired*	334,089	35.45	35.22–35.68	44.28	43.82–44.74	55.72	55.26–56.18	
*Out of work*	30,263	4.56	4.44–4.67	48.35	47.89–48.81	51.65	51.19–52.11	
*Unable to work*	68,598	9.29	9.14–9.43	57.79	57.41–58.17	42.21	41.83–42.59	
Education								
*Less than high school*	46,057	11.32	11.12–11.51	70.25	69.64–70.87	29.75	29.13–30.36	<0.0001
*High school graduate*	212,952	26.89	26.68–27.11	32.53	32.22–32.85	67.47	67.15–67.78	
*Some college/technical school*	231,936	31.23	31.00–31.47	37.83	37.53–38.14	62.17	61.86–62.48	
*College graduate or more*	331,838	30.56	30.35–30.77	49.81	49.56–50.07	50.19	49.93–50.44	
Marital Status								
*Married/unmarried couple*	493,189	63.52	63.28–63.75	51.23	50.92–51.55	48.77	48.45–49.08	<0.0001
*Never married*	74,175	8.62	8.48–8.76	44.74	43.90–45.58	55.26	54.42–56.10	
*Separated/divorced/widowed*	255,419	27.87	27.65–28.09	46.51	46.06–46.95	53.49	53.05–53.94	
Self-Reported Health Status								
*Excellent*	132,067	15.97	15.79–16.15	46.58	45.96–47.20	53.42	52.80–54.04	<0.0001
*Very good*	281,139	32.06	31.83–32.29	50.57	50.15–50.99	49.43	49.01–49.85	
*Good*	257,381	31.91	31.67–32.14	49.50	49.05–49.95	50.50	50.05–50.95	
*Fair*	112,983	14.95	14.77–15.13	49.29	48.62–49.95	50.72	50.05–51.38	
*Poor*	39,213	5.11	5.00–5.22	49.76	48.67–50.85	50.24	49.15–51.33	
Angina/Coronary Heart Disease								
*Yes*	57,055	6.50	6.39–6.62	58.56	57.67–59.45	41.44	40.55–42.33	<0.0001
*No*	765,728	93.50	93.38–93.61	48.72	48.46–48.98	51.28	51.03–51.54	
Diabetes								
*Yes*	148,547	18.66	18.46–18.86	57.70	57.12–58.29	42.30	41.71–42.88	<0.0001
*No*	674,236	81.34	81.15–81.54	47.44	47.17–47.72	52.56	52.28–52.83	
Smoking Status								
*Current smoker*	116,438	14.89	14.71–15.05	37.42	36.84–38.01	62.58	61.99–63.16	<0.0001
*Former smoker*	255,735	30.22	30.00–30.45	53.28	52.85–53.71	46.72	46.29–47.15	
*Never smoker*	450,610	54.89	54.65–55.14	50.43	50.09–50.78	49.57	49.22–49.91	

**Table 2:** Weighted unadjusted/adjusted logistic regression results of SIV by HCOPD within subgroups.

	Total Unweighted n	Unweighted n with Outcome (Flu Shot) (Unweighted Count, Weighted Prevalence)	Weighted/Unadjusted		Weighted/Adjusted	
	#	#	WUOR	95% CI	WAOR	95% CI
**Overall**	822,783	431,990 (49.36%)	1.27	1.23, 1.32 ****	1.23	1.19, 1.28 ****
**Current Smoker**	116,438	46,979 (37.42%)	1.63	1.54, 1.73 ****	1.36	1.28, 1.45 ****
**Male**	56,059	20,593 (33.57%)	1.69	1.55, 1.84 ****	1.35	1.23, 1.48 ****
**2017**	10,310	3,653 (33.29%)			1.33	1.09, 1.62 **
**2018**	10,628	3,111 (27.22%)			1.19	0.98, 1.45 ^+^
**2019**	9,035	3,312 (32.63%)			1.55	1.27, 1.88 ****
**2020**	8,890	3,473 (35.39%)			1.52	1.22, 1.88 ***
**2021**	8,764	3,573 (37.27%)			1.24	0.96, 1.60 ^+^
**2022**	8,432	3,471 (37.09%)			1.30	1.07, 1.58 ***
**Female**	60,379	26,386 (41.69%)	1.49	1.38, 1.61 ****	1.31	1.20, 1.43 ****
**2017**	11,603	4,897 (39.36%)			1.26	1.04, 1.52 *
**2018**	11,137	3,732 (32.60%)			1.23	1.03, 1.48 *
**2019**	9,975	4,468 (42.33%)			1.42	1.19, 1.70 ****
**2020**	9,410	4,517 (46.16%)			1.28	1.01, 1.64 *
**2021**	9,479	4,554 (45.60%)			1.25	1.02, 1.53 *
**2022**	8,775	4,218 (45.40%)			1.33	1.10, 1.61 **
**Former Smoker**	255,735	185,716 (53.28%)	1.50	1.43, 1.58 ****	1.35	1.27, 1.43 ****
**Male**	132,033	71,828 (51.78%)	1.70	1.58, 1.83 ****	1.45	1.33, 1.58 ****
**2017**	24,367	12,577 (50.62%)			1.25	1.01, 1.54 *
**2018**	24,464	10,993 (42.66%)			1.24	1.04, 1.47 *
**2019**	21,414	11,921 (52.31%)			1.70	1.41, 2.07 ****
**2020**	20,000	11,692 (54.56%)			1.91	1.47, 2.49 ****
**2021**	21,037	12,349 (56.44%)			1.11	0.93, 1.32
**2022**	10,751	12,296 (55.83%)			1.74	1.44, 2.11 ****
**Female**	123,702	71,391 (55.13%)	1.31	1.22, 1.40 ****	1.24	1.15, 1.35 ****
**2017**	23,640	12,752 (51.71%)			1.55	1.31, 1.84 ****
**2018**	22,875	10,662 (44.68%)			1.34	1.10, 1.63 **
**2019**	19,852	11,685 (56.51%)			1.23	1.04, 1.47 *
**2020**	18,744	11,825 (60.83%)			1.15	0.94, 1.41
**2021**	19,210	12,175 (59.45%)			1.06	0.87, 1.30
**2022**	19,381	12,292 (58.83%)			1.14	0.94, 1.38
**Never Smoker**	450,610	241,792 (50.43%)	1.26	1.17, 1.36 ****	1.18	1.09, 1.27 ****
**Male**	191,370	96,015 (47.42%)	1.38	1.19, 1.60 ****	1.23	1.05, 1.44 ****
**2017**	32,024	15,216 (43.71%)			1.45	1.07, 1.97 *
**2018**	32,953	13,687 (39.00%)			1.39	1.03, 1.89*
**2019**	30,571	15,388 (47.18%)			1.30	0.95, 1.78
**2020**	30,076	16,271 (51.06%)			1.14	0.65, 1.99
**2021**	32,479	18,012 (51.83%)			1.16	0.87, 1.53
**2022**	33,267	18,441 (51.47%)			0.99	0.74, 1.33
**Female**	259,240	144,777 (52.93%)	1.16	1.07, 1.25 ***	1.13	1.04, 1.23 **
**2017**	47,906	25,055 (48.91%)			1.22	1.01, 1.48*
**2018**	46,405	21,253 (43.50%)			1.10	0.91, 1.34
**2019**	41,911	23,504 (51.73%)			1.04	0.86, 1.26
**2020**	40,420	24,539 (57.35%)			1.03	0.82, 1.31
**2021**	41,569	25,365 (58.05%)			1.18	0.95, 1.46
**2022**	41,029	25,061 (58.40%)			1.24	0.99, 1.54 ^+^

WUOR: Weighted Unadjusted Odds Ratio; WAOR: Weighted Adjusted Odds Ratio controlling for age, race, education, employment, income, marital status, self-reported health status, health insurance, CHD, and diabetes; 95% CI: 95% Confidence Intervals for Odds Ratios; ⁺ *p* < 0.10; * *p* < 0.05; ** *p* < 0.01; *** *p* < 0.001; **** *p* < 0.0001.

## Data Availability

All BRFSS data are publicly available.
